# Reevaluating the Role of the Hippocampus in Delay Eyeblink Conditioning

**DOI:** 10.1371/journal.pone.0071249

**Published:** 2013-08-09

**Authors:** Guang-yan Wu, Juan Yao, Bo Hu, Hui-ming Zhang, Yi-ding Li, Xuan Li, Qiong Li, Jian-feng Sui

**Affiliations:** 1 Department of Physiology, College of Basic Medical Sciences, Third Military Medical University, Chongqing, China; 2 Experimental Center of Basic Medicine, College of Basic Medical Sciences, Third Military Medical University, Chongqing, China; Radboud University, The Netherlands

## Abstract

The role of the hippocampus in delay eyeblink conditioning (DEC) remains controversial. Here, we investigated the involvement of the hippocampus in DEC with a soft tone as the conditioned stimulus (CS) by using electrolytic lesions or muscimol inactivation of guinea pig dorsal hippocampus. Interestingly, when a soft tone was used as a CS, electrolytic lesions of the hippocampus significantly retarded acquisition of the conditioned response (CR), and muscimol infusions into hippocampus distinctly inhibited the acquisition and expression of CR, but had no significant effect on consolidation of well-learned CR. In contrast, both electrolytic lesions and muscimol inactivation of hippocampus produced no significant deficits in the CR when a loud tone was used as the CS. These results demonstrate that the hippocampus is essential for the DEC when the delay task was rendered more difficult.

## Introduction

Classical eyeblink conditioning provides exciting opportunities for investigating the neural substrates and mechanisms underling associative learning and memory [1]–[9], which involves paired presentations of a behaviorally neutral conditioned stimulus (CS; e.g., a tone or light) and an unconditioned stimulus (US; e.g., a corneal airpuff or periorbital shock). Initially, the organism produces only a reflexive eyeblink unconditioned response (UR) to the US. After hundreds of paired presentations of the CS and the US, the organism could learn to close the eyes in response to the CS before the onset of the US, which is called the conditioned response (CR). According to the temporal relationship between the CS and the US, there are two commonly used procedures in eyeblink conditioning: trace and delay paradigms. In the trace eyeblink conditioning (TEC), a temporal gap occurs between the offset of the CS and the onset of the US, which is in contrast to the delay eyeblink conditioning (DEC), in which the CS overlaps the US and the two stimuli are terminated at the same time [8], [10].

In TEC, several forebrain structures, such as the hippocampus [11]–[14], medial prefrontal cortex (mPFC) [15]–[20], and ventrolateral thalamic nuclei [21], are required for learning, in addition to a brainstem-cerebellar circuit. In contrast to the TEC, it is mostly established that the brainstem-cerebellar circuit is essential and sufficient for the simple DEC [1], [3], [6], [22]–[24]. Interestingly, while cumulative evidence has demonstrated that the hippocampus is not critical for simple DEC [11], [25]–[27], the CA1 field of the hippocampus shows increases in frequency of pyramidal cell firing and in 2-deoxyglucose during simple DEC [23], [24], [28], [29], and the hippocampus may play a role in the retention of DEC [30] and in the acquisition of long DEC with 1400-ms interstimulus intervals (ISI; the interval between the CS and US onsets) [11]. Similarly, although the mPFC is reportedly not critical for DEC [6], [17], [18], [20], [31]–[36], lesions of mPFC impaired the DEC with a relative low-intensity tone CS (soft CS, e.g., 60 dB) [37] and the mPFC shows increases in the marker of metabolic activity 2-deoxyglucose during DEC [24]. In addition, the ventrolateral thalamic nuclei may not be necessary for standard DEC, whereas it may be involved in regulating the acquisition and/or performance of the DEC with non-optimal training parameters (e.g., 1000-ms ISI) [21]. These findings, taken as a whole, demonstrate that the forebrain structures (e.g., hippocampus) may play potential roles in DEC. However, there is no convincing evidence to support the hypothesis that the hippocampus is critical for DEC.

It is often stated that the hippocampus and mPFC are involved in “filling the gap” or otherwise associating the CS and US in time [6], [7], [11], [38]. However, in addition to the presence of a temporal gap, there are other differences between TEC and DEC. The most apparent difference is that animals require much more training to learn the TEC, and thus it is inherently more difficult than the DEC [39], [40]. Indeed, when the DEC was rendered more difficult by extending the ISI (long delay conditioning), hippocampal lesions impaired acquisition of the DEC [11]. Furthermore, our previous work suggests that electrolytic lesions or muscimol inactivation of mPFC has significant effect on the DEC with a relative low-intensity tone CS [37], which is comparably difficult for the animal to learn. These findings and similar ones led us to propose the hypothesis that like the case of mPFC, the hippocampus is essential for the DEC with a soft tone CS but not for the DEC with a loud tone CS.

Here, we investigated the involvement of the hippocampus in the DEC with a soft tone (60 dB, 2 kHz) or loud tone (85 dB, 2 kHz) CS. The present results provide direct support for our original hypothesis and suggest that the hippocampus is essential for the DEC with the soft tone CS but not for the DEC with the loud tone CS.

## Materials and Methods

### Ethics Statement

The experimental procedures were approved by the Animal Care Committee of the Third Military Medical University and were in accordance with the principles outlined in the NIH Guide for the Care and Use of Laboratory Animals. All possible efforts were made to optimize the comfort and to minimize the use of the animals.

### Subjects

A total of 64 adult female albino Dunkin-Hartley guinea pigs, weighing 500–550 g (4–5 months old) at the time of surgery, were included in the study. Before the experiments and between the conditioning sessions, these animals were individually housed in standard plastic cages on a 12∶12 light/dark cycle with free access to food and water *ad libitum*. The room temperature was maintained at 25±1°C. All of the experiments were performed between 8∶00 A.M. and 6∶00 P.M., during the light portion of the cycle.

The guinea pigs were divided into eight conditioning groups [i.e., Soft-Lesion (n = 8), Soft-Sham (n = 8), Soft-MAMA (n = 8), Soft-AAAA (n = 8), Loud-Lesion (n = 8), Loud-Sham (n = 8), Loud-Muscimol (n = 8), and Loud-ACSF (n = 8) groups], according to the loudness level of the tone (a soft or loud tone) CS, the type of the damage (electrolytic lesion or muscimol inactivation), and the variety and sequence of the infused substances. It is worth asking whether hippocampus is involved in both acquisition, expression, and consolidation processes in DEC with the soft tone CS. Thus, the Soft-MAMA and Soft-AAAA groups passed through four phases, and were infused with either muscimol or artificial cerebrospinal fluid (ACSF) for 20 sessions (phase I); both ACSF for 8 sessions (phase II), either muscimol or ACSF for 1 session (phase III), and both ACSF for 1 session (phase IV). Moreover, “MAMA” represents muscimol-ACSF-muscimol-ACSF infusion, and “AAAA” represents ACSF-ACSF-ACSF-ACSF infusion, corresponding to phase I, II, III, and IV respectively in each group.

### Surgery

The surgical procedures for eyeblink recording were conducted essentially as described by Wu et al. [37]. In brief, all animals were fitted with a headstage and a loop attached to the apex of the left upper eyelid. In the current study, this loop was utilized to attach the left upper eyelid to a movement-measuring device. Moreover, for each animal in Soft-MAMA, Soft-AAAA, Loud-Muscimol, and Loud-ACSF groups, four guide cannulae (No. 62001, RWD, Shenzhen, China) were implanted into the bilateral dorsal hippocampus. Four stylets (No. 62101, RWD, Shenzhen, China) were inserted into the guide cannulae and extended 0.5 mm beyond the tips of the guide cannula. The stereotaxic coordinates were 6.2 and 5.4 mm anterior to frontal zero plane, ±2.0 and ±5.0 mm lateral to midline, and 4.5 and 5.2 mm below the skull surface. For each animal in Soft-Lesion and Loud-Lesion groups, the bilateral dorsal hippocampus were given electrolytic lesions, produced by passing 3.0 mA of DC current for 20 s at the four sites via custom-made electrodes, which were made of PFA-Insulated stainless steel wire (NO. 792500, A-M Systems, Sequim, WA, USA; coated diameter: 330.20 µm, bare diameter: 254.00 µm). These parameters of electrical lesions were chosen based on a recent study [15]. The stereotaxic coordinates were 6.2 and 5.4 mm anterior to frontal zero plane, ±2.0 and ±5.0 mm lateral to midline, and 4.5 and 5.2 mm below the skull surface. After the surgery, the animals were allowed 1 week of recovery.

### Apparatus

**Figure 1:**
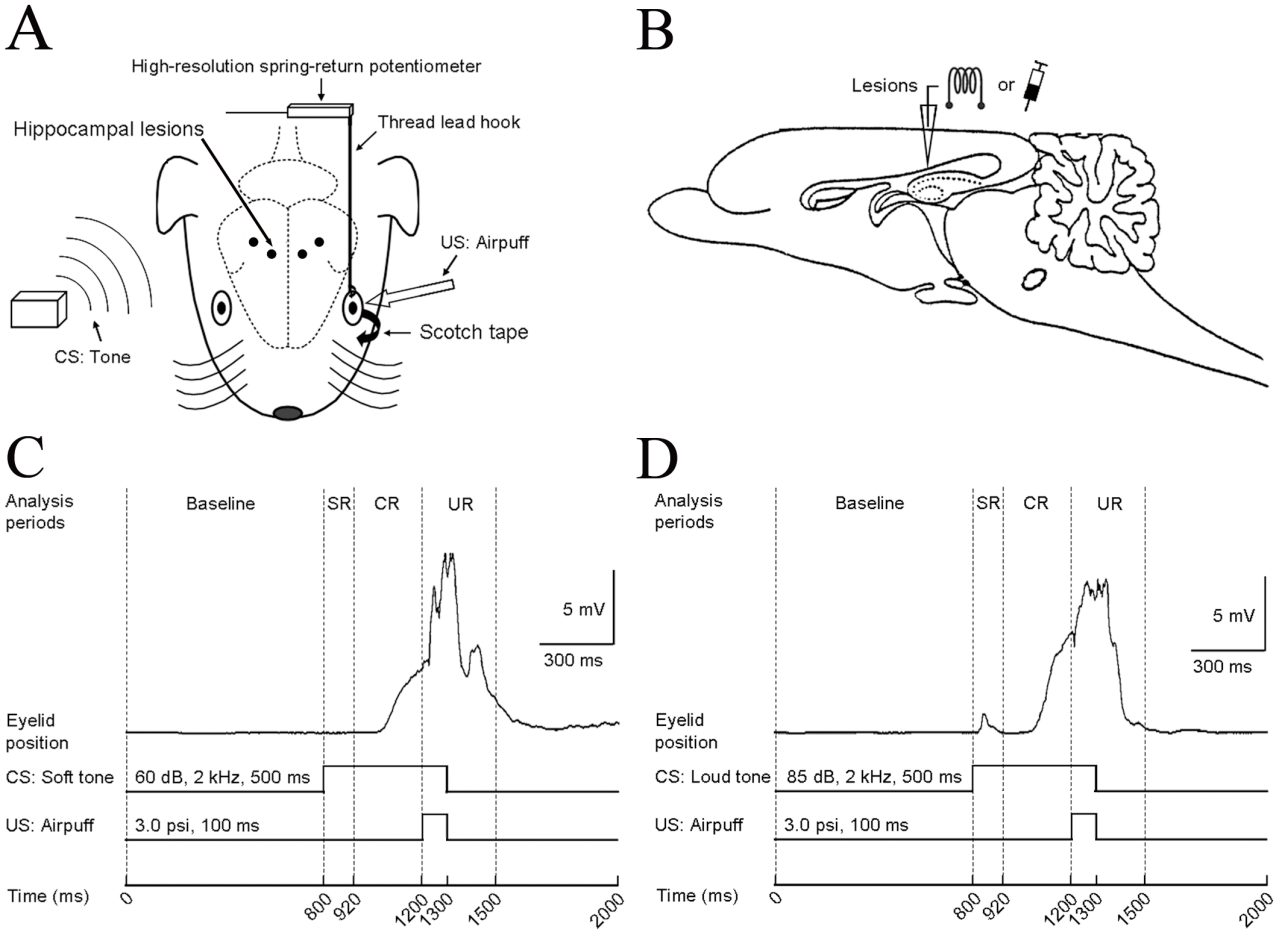
Experimental design. (A) The upper left eyelid movements were measured by a high-resolution spring-return potentiometer that was attached via a thread lead that was hooked through a nylon loop, which was sutured into the left upper eyelid, and the left lower eyelid was taped open. Two loudness levels of tone (i.e., 60 dB or 85 dB) were presented binaurally, as a soft or loud tone conditioned stimulus (CS), and airpuffs were presented to the ipsilateral cornea as an unconditioned stimulus (US). Moreover, electrolytic lesions or muscimol inactivation were performed in the guinea pigs’ bilateral dorsal hippocampus. Four black circles represent four lesion sites. (B) Diagram of the sagittal section of guinea pig brain, showing the lesion electrode or infusion sites. (C, D) The temporal relationship of the CS, US and analysis periods during delay eyeblink conditioning with a soft (C) and loud (D) tone CS. In each trial, we analyzed the parameters of the startle eyeblink response (SR; 0–120 ms period after the CS onset), conditioned eyeblink response (CR; 120–400 ms period after the CS onset) and unconditioned eyeblink response (UR; 0–300 ms period after the US onset). These responses were based on the average magnitude of the baseline (a 0–800 ms period prior to the onset of the CS). Examples of two typical SR, CR and UR from two records were exhibited during delay eyeblink conditioning with the soft (C) and loud (D) tone CS.

Eyelid movements were measured by a high-resolution spring-return potentiometer (JZ101, XH, Beijing, China) that was attached via a thread lead that was hooked through the nylon loop that was sutured into the left upper eyelid. A speaker that was placed 60 cm above the animal was used to deliver either a soft or a loud tone CS, while a plastic pipe placed 1.0 cm from the animal’s left eyeball was used to deliver a corneal airpuff US. Presentations of the CS and US were controlled by a homemade computer-monitored system (Figure 1A). The eyelid movement mechanogram and markers of the applied stimuli were digitized at a sample rate of 10 kHz by a data acquisition system (RM6280C, Cheng Yi, Chengdu, China) and were acquired using the built-in software (v. 4.7). A Windows PC was used to store the behavioral data.

### Behavioral Procedures

Following postoperative recovery, all animals were adapted to the experimental environment for two sessions, with 90 min/session. These two sessions were followed by daily sessions of delay eyeblink conditioning. During all of the experimental sessions, the animals were restrained in a plexiglas container (25 × 15 × 15 cm) that was located in a sound- and light-attenuating chamber, and their heads were secured with blunt earbars pressing on the headstages. The left eye of the animal was held open in a confirmable position, with the nylon loop sutured into the left upper eyelid, which was linked to the high-resolution spring-return potentiometer. The voltage level represented the eyeblink baseline position, which was manually calibrated to a constant value. Moreover, the animal’s left lower eyelid was taped open. These two measures were made to insure continual exposure of the left cornea.

The animals were trained in the delay conditioning paradigm. The CS was a 500-ms, 2-kHz pure tone with either 60 dB or 85 dB (range in conditioning chamber, 58–62 dB or 83–87 dB; tested by sound level meter, type 2240, Brüel & Kjær, Denmark), corresponding to soft or loud tone CS respectively. The basal sound level was approximately 35 dB. The US was a 3.0-psi (measured at the tip of the plastic pipe), 100-ms corneal airpuff. During all of the CS-US paired trials, the CS terminated simultaneously with the US. The daily conditioning session (day) consisted of ten 10-trial blocks, each of which comprised nine CS-US paired trials and one CS-alone trial. The trials were separated by a variable intertrial interval of 20–40 s (with a mean of 30 s). The animals of the Soft-lesion and Soft-sham groups were sequentially trained on the DEC with the soft tone CS for 20 sessions, and the animals of the Loud-Lesion, Loud-Sham, Loud-Muscimol, and Loud-ACSF groups were sequentially trained on the DEC with the loud tone CS for 6 sessions. Because DEC with a soft tone CS is a more difficult task than that with a loud tone CS, and animals learned the DEC with a loud tone CS much more quickly than that with a soft tone CS. Thus, the training sessions for loud were 6 vs. for soft were 20. Furthermore, the animals of the Soft-MAMA and the Soft-AAAA groups were sequentially trained on the DEC with the soft tone CS after daily infusion of either muscimol or ACSF for 20 sessions (phase I); both ACSF for 8 sessions (phase II), either muscimol or ACSF for 1 session (phase III), and both ACSF for 1 session (phase IV).

### Drug Microinfusions

The GABA_A_ receptor agonist muscimol (Sigma-Aldrich, St. Louis, MO, USA) was dissolved in ACSF consisting of (in mM): 126 NaCl, 5 KCl, 1.25 NaH_2_PO_4_, 2 MgSO_4_, 26 NaHCO_3_, 2 CaCl_2_, and 10 glucose (pH 7.35–7.40). According to their group assignments and phase sequences, the guinea pigs of the Soft-MAMA and Soft-AAAA groups were infused with 0.5 µl of muscimol (1.0 mM, pH 7.35–7.40) or 0.5 µl of ACSF into the every site (two sites on each side) 30 min before the daily conditioning training. Infusion procedures for each animal included removal of the internal stylet from the guide cannula, insertion of a stainless steel infusion cannula (No. 62201, RWD, Shenzhen, China; external diameter: 0.20 mm, internal diameter: 0.10 mm) that extended 0.5 mm below the tip of the guide cannula, infusion of the drug at 0.25 µl/min via polyethylene tubing connected to a microsyringe, removal of the infusion cannula 5 min after the cessation of infusion, and finally reinsertion of the internal stylet.

### Histology

After the completion of the behavioral experiments, all of the animals except the animals of Soft-Sham and Loud-Sham groups were given a lethal dose of pentobarbital sodium (150 mg/kg, i.p.;SCRC, Shanghai, China) and were perfused transcardially with physiological saline followed by 4% paraformaldehyde (prepared in 0.1 M of phosphate buffer, pH 7.35–7.40). The brains were removed from the skull and stored in 4% paraformaldehyde for several days. Four days prior to sectioning, the brains were transferred to a 30% sucrose/4% paraformaldehyde solution. Frozen coronal sections of 20-µm thickness were taken through the sites of the electrolytic lesion and guide cannula implantation. The slices were stained with cresyl violet. The extents of electrolytic lesions and locations of the infusion cannula tips within the brains were carefully checked using a light microscope (SMZ1500, Nikon, Tokyo, Japan) with a digital camera (DXM1200F, Nikon, Tokyo, Japan) and were drawn onto plates from the stereotaxic atlas of the guinea pig brain [41].

### Behavioral Data Analysis

A detailed description of eyeblink response analysis was previously described by Wu et al. [37]. Briefly, each trial was subdivided into four continuous analysis periods: (1) the “baseline” period, 0–800 ms before the CS onset; (2) the “eyeblink startle response (SR)” period, 0–120 ms after the CS onset; (3) the “CR” period, 120–400 ms after the CS onset; (4) the “UR” period, 0–300 ms after the US onset (Figure 1C and D). A significant eyelid movement was defined as an increase in the mechanogram magnitude that was greater than the mean baseline magnitude, plus four times the standard deviation of the baseline activity. In addition, a significant eyelid movement was also required to have a minimal duration of 15 ms and to exceed the 1 mV baseline threshold (equaled 0.25 mm). Any significant eyelid movement during the above-mentioned periods was counted as an SR, a CR or a UR. The magnitude, onset latency, and peak latency of the CR were measured on the trials in which a CR occurred.

### Statistical Analysis

All of the data were expressed as the mean ± SEM. The statistical significance was determined by the least significant difference (LSD) post hoc tests, following a two-way repeated measures analyses of variance (ANOVA), a separate one-way repeated measures ANOVA, or a separate one-way ANOVA, or by an independent-samples t test using the SPSS software for the Windows package (v. 18.0). A value of *p*<0.05 was considered to be statistically significant.

## Results

### The Extents of Electrolytic Lesions and the Placements of Infusion Cannula Tips

**Figure 2:**
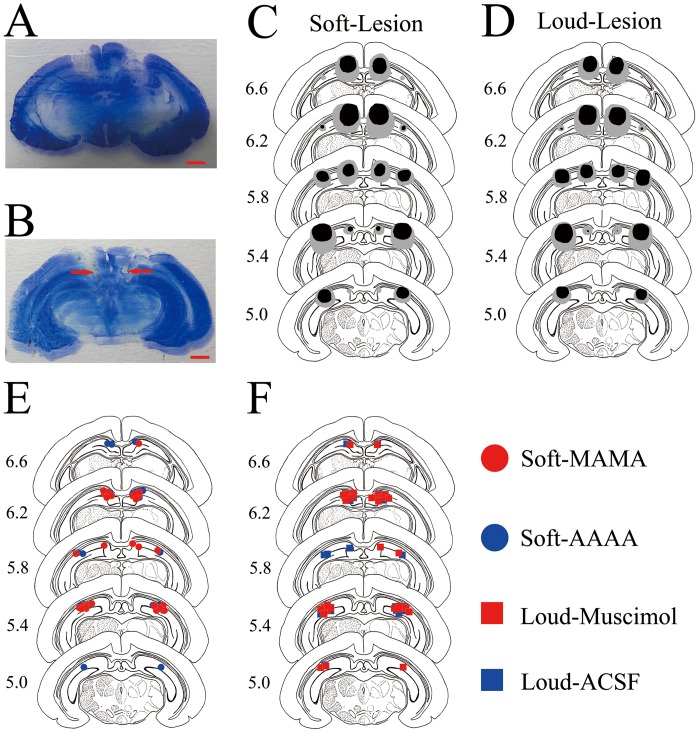
Histological reconstructions of the extents of electrolytic lesions and the placements of infusion cannula tips. (A, B) Two photomicrographs of two coronal sections showing a representative extent of electrolytic lesion of the dorsal hippocampus and a representative placement of infusion cannula tip in the dorsal hippocampus, respectively. Scale bars represent 2 mm. Red arrows indicate the locations of the infusion cannula tips. (C, D) The largest (gray shaded areas) and smallest (black shaded areas) lesions of the dorsal hippocampus in the Soft-Lesion (n = 7, C) and Loud-Lesion (n = 7, D) groups. (E, F) Schematic illustration of all infusion cannula tips placements in the Soft-MAMA (n = 8), Soft-AAAA (n = 8) (E), Soft-Muscimol (n = 8), and Soft-ACSF (n = 8) groups (F). A red circle, blue circle, red square, or blue square represents a placement of infusion cannula tip in the Soft-MAMA, Soft-AAAA, Soft-Muscimol, or Soft-ACSF group, respectively. Numbers to the left represent distance (mm) from the frontal zero plane. The coronal brain plates are adapted from the atlas of Rapisarda and Bacchelli (1977).

We carefully checked the extents of hippocampal lesions in the Soft-Lesion and Loud-Lesion groups before the behavioral analysis. The data from one animal in the Loud-Lesion group and from one animal in the Soft-Lesion group were excluded from analysis because they died before the end of the experiment. As shown in Figure 2C and D, the largest and smallest lesions in the Soft-Lesion (n = 7, C) and Loud-Lesion (n = 7, D) groups were mainly focused on the hippocampus. Figure 2A is a representative photomicrograph of the hippocampal lesions. Similarly, the placements of infusion cannula tips were also carefully checked before the behavioral analysis. The data from an animal were excluded from the analysis if any infusion cannula tip was not in or near the hippocampus. As seen in Figure 2E and F, All of the infusion cannula tips placements of the Soft-MAMA (n = 8, E), Soft-AAAA (n = 8, E), Loud-Muscimol (n = 8, F), and Loud-ACSF (n = 8, F) groups were in the hippocampus. Figure 2B is a representative photomicrograph of an infusion cannula tip placement in the hippocampus.

### Effects of the Electrolytic Lesions of the Hippocampus on the Delay Eyeblink Conditioning with a Soft Tone CS

**Figure 3:**
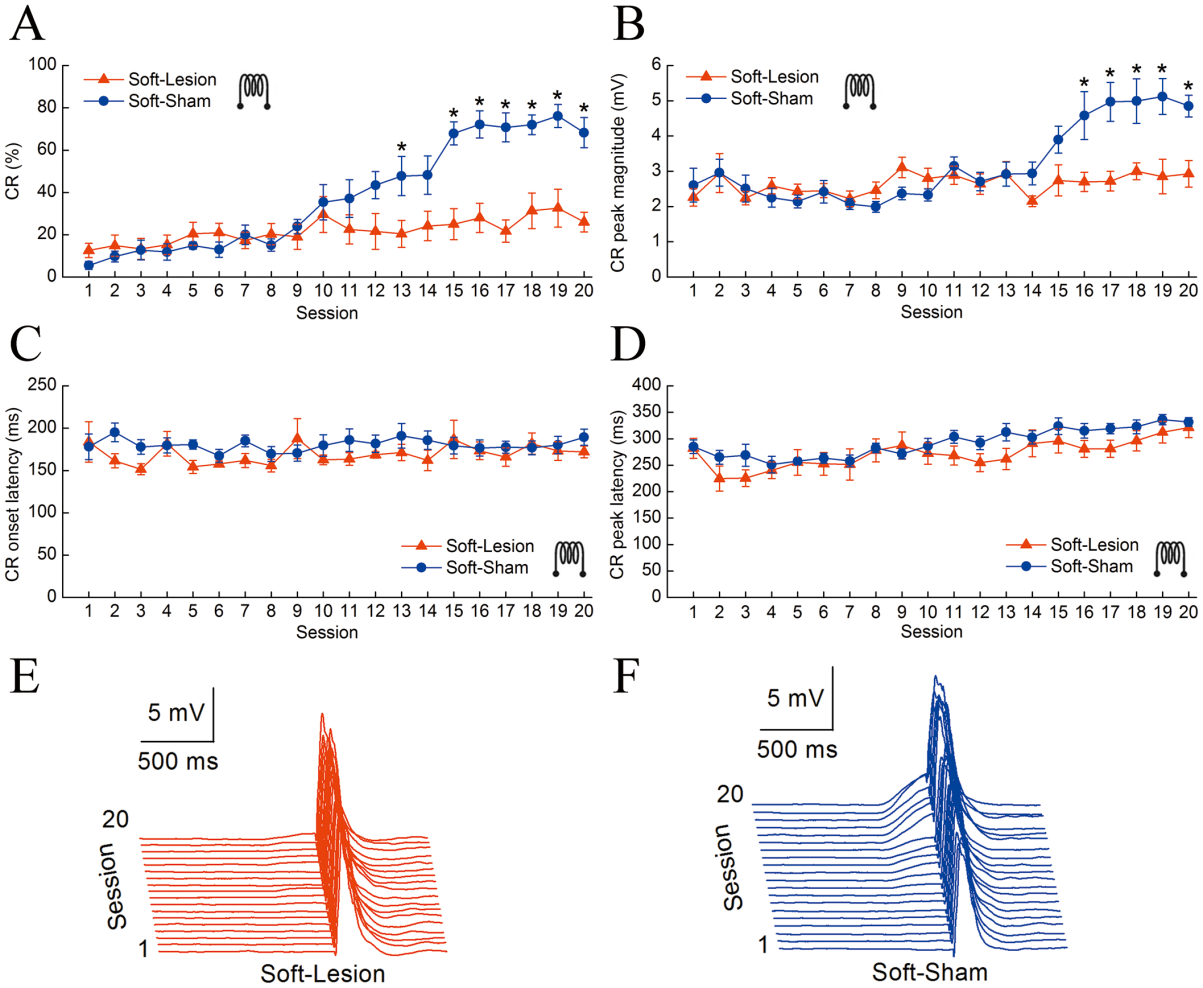
Effects of electrolytic lesions of the hippocampus before conditioning training on delay eyeblink conditioning with the soft tone CS. (A–D) The mean value ± standard error (SEM) for the percentage (A), peak magnitude (B), onset latency (C), and peak latency (D) of CR with the soft tone CS in the Soft-lesion (n = 7, red) and Soft-sham (n = 8, blue) groups. (E, F) Averaged eyelid responses of all trials in the Soft-lesion (E) and Soft-sham (F) animals across twenty consecutive training sessions. Note that there were only significant differences in the percentage and peak magnitude of the CR between the Soft-lesion and Loud-Lesion groups. *p<0.05 versus control. The error bars represent the SEM.

Figure 3 shows the effects of the electrolytic lesions and sham lesions of the bilateral hippocampus on the DEC with the soft tone CS before the conditioning training in the Soft-Lesion (n = 7) and Soft-Sham (n = 8) groups. The data from one animal in the Soft-Lesion group were removed from the analysis because it died before the end of the experiment. As shown in Figure 3A, guinea pigs with hippocampal lesions were significantly impaired in acquisition of the DEC with the soft tone CS relative to the sham animals. This result was confirmed by performing a two-way repeated measure ANOVA on the CR%. There was a significant group by session interaction [*F*(19,247) = 13.650, *p*<0.001], and significant effects of the group [*F*(1,13) = 6.315, *p* = 0.026] and the session [*F*(19,247) = 27.197, *p*<0.001]. Furthermore, a separate one-way ANOVA revealed that the CR% of the Soft-Sham group was significantly higher than that of the Soft-Lesion group on session 13 [*F*(1,13) = 5.579, *p* = 0.034] and sessions 15–20 [*F*(1,13) = 22.930, *p*<0.001; *F*(1,13) = 22.002, *p*<0.001; *F*(1,13) = 30.898, *p*<0.001; *F*(1,13) = 19.103, *p* = 0.001; *F*(1,13) = 18.337, *p* = 0.001; and *F*(1,13) = 23.143, *p*<0.001, respectively].

To investigate the effects of electrolytic lesions of the bilateral hippocampus on the pattern of the CR with the soft tone CS, we analyzed the CR peak magnitude, onset latency, and peak latency for these animals. The CR peak magnitude across the 20 sessions is illustrated in Figure 3B. The Soft-Sham animals had significantly greater CR peak magnitude than Soft-Lesion animals [two-way repeated measures ANOVA; *F*(1,13) = 5.292, *p* = 0.039, respectively]. Additionally, the CR onset latency and peak latency across the 20 sessions are shown in Figure 3C and D. There were no significant differences in the CR onset latency or peak latency between the Soft-Lesion and Soft-Sham groups [two-way repeated measures ANOVA; *F*(1,13) = 2.361, *p* = 0.148; *F*(1,13) = 3.395, *p* = 0.088; respectively].

### Effects of the Infusion of Muscimol into the Hippocampus on the Delay Eyeblink Conditioning with a Soft Tone CS

**Figure 4:**
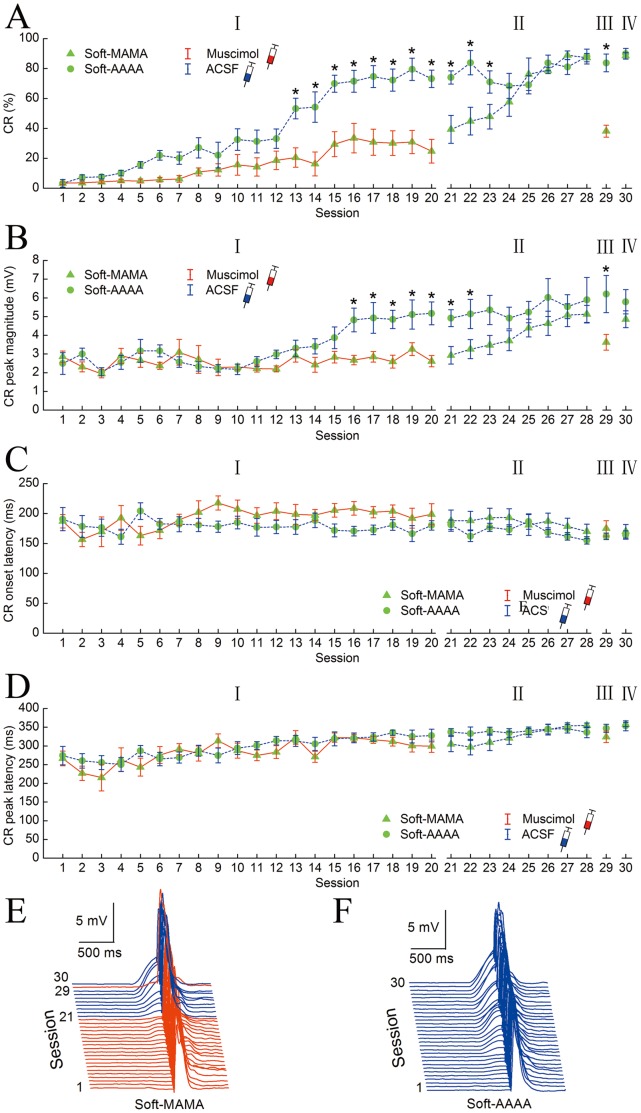
Effects of the infusion of muscimol into the hippocampus 30 min before daily conditioning training during the inactivation phases (phases I and III) on delay eyeblink conditioning with the soft tone CS. MAMA, muscimol-ACSF-muscimol-ACSF infusion; AAAA, ACSF-ACSF-ACSF-ACSF infusion. A solid red line and dash blue line represents muscimol or PBS infusion, respectively. (A–C) The mean value ± standard error (SEM) for the percentage (A), peak magnitude (B), onset latency (C), and peak latency (D) of the CR with the soft tone CS in the Soft-MAMA (n = 8, green triangle) and Soft-AAAA (n = 10, green circle) groups. (E, F) Averaged eyelid responses of all trials in the Soft-MAMA (E) and Soft-AAAA (F) animals across thirty consecutive training sessions. Note that there were only significant differences in the percentage and peak magnitude of the CR between the Soft-MAMA and Soft-AAAA groups in phases I, II and III. *p<0.05 versus control. The error bars represent the SEM.

To specify the involvement of the hippocampus in the explicit processes of the delay CR with the soft tone CS, with respect to acquisition, consolidation, storage, and expression, we investigated the effects of the muscimol infusion into the bilateral hippocampus 30 min before daily training during the inactivation phases (phases I and III) on the DEC with the soft tone CS. Figure 4 shows the effects on the DEC with the soft tone CS of an infusion of muscimol or ACSF into the bilateral hippocampus before the daily conditioning training in the Soft-MAMA (n = 8) and Soft-AAAA (n = 8) groups. Phase I (sessions 1–20) was designed to determine the effects of muscimol inactivation of hippocampus on the acquisition of the delay CR with the soft tone CS. As expected, a two-way repeated measures ANOVA on the CR% (Figure 4A) revealed that there was a significant group by session interaction [*F*(19,266) = 5.330, *p*<0.001], and significant effects of the group [*F*(1,14) = 15.704, *p* = 0.001] and the session [*F*(19,266) = 26.683, *p*<0.001] in phase I. Furthermore, a separate one-way ANOVA revealed that the CR% of the Soft-AAAA group was significantly higher than that of the Soft-MAMA group on sessions 13–20 [*F*(1,14) = 10.001, *p* = 0.007; *F*(1,14) = 8.633, *p* = 0.011; *F*(1,14) = 8.450, *p* = 0.011; *F*(1,14) = 7.627, *p = *0.015; *F*(1,14) = 11.855, *p* = 0.004; *F*(1,14) = 12.620, *p* = 0.003; *F*(1,14) = 25.429, *p*<0.001; and *F*(1,16) = 24.257, *p*<0.001, respectively]. In phase II (sessions 21–28), the Soft-MAMA group significantly increased its CR%, and the CR% of both groups reached an asymptotic level (Figure 4A). A two-way repeated measures ANOVA on the CR% revealed that there was a significant group by session interaction [*F*(7,98) = 8.792, *p*<0.001], and significant effects of the groups [*F*(1,14) = 6.483, *p* = 0.037] and the sessions [*F*(7,98) = 16.076, *p*<0.001] in phase II. Moreover, a separate one-way ANOVA that the CR% of the Soft-AAAA group was significantly higher than that of the Soft-MAMA group on sessions 21–23 [*F*(1,14) = 14.815, *p* = 0.002; *F*(1,14) = 12.011, *p* = 0.004 and *F*(1,14) = 4.972, *p* = 0.043, respectively]. Phase III (session 29) was designed to examine the effects of muscimol inactivation of hippocampus on the expression of the delay CR with the soft tone CS. The Soft-MAMA group significantly reduced its CR% relative to the Soft-AAAA group in phase III (Figure 4A). A independent-samples t test on the CR% revealed that the CR% of the Soft-AAAA group was significantly higher than that of the Soft-MAMA group in phase III (*t = *6.213, *p*<0.001). Finally, phase IV (session 30) was designed to examine the effects of the hippocampal inactivation in phase III on the consolidation of the well-learned delay CR with the soft tone CS. An independent-samples t test on the CR% revealed that Soft-MAMA group did not differ significantly from Soft-AAAA group (*t = *0.464, *p* = 0.649) (Figure 4A).

To investigate the effects of infusion of muscimol into the bilateral hippocampus on the pattern of the CR with the soft tone CS, we analyzed the CR peak magnitude, onset latency, and peak latency for these animals. The CR peak magnitude across 30 sessions is shown in Figure 4B. The CR peak magnitude of the Soft-MAMA group was significantly lower than that of the Soft-AAAA group in phase I, II, and III [two-way repeated measures ANOVA or independent-samples t test; *F*(1,14) = 5.084, *p* = 0.041; *F*(1,14) = 2.875, *p* = 0.112; and *t = *2.384, *p* = 0.032; respectively]. However, there were no significant differences in the CR peak magnitude between the Soft-MAMA and Soft-AAAA groups in phase IV (independent-samples t test; *t = *1.173, *p* = 0.260). Furthermore, the CR onset latency and peak latency across the 30 sessions are depicted in Figure 4C and D. The CR onset latency and peak latency of the Soft-MAMA did differ significantly from those of Soft-AAAA groups in phases I – IV (two-way repeated measures ANOVA or independent-samples t test, all *P>*0.083).

### Effects of Electrolytic Lesions of the Hippocampus on the Delay Eyeblink Conditioning with a Loud Tone CS

**Figure 5:**
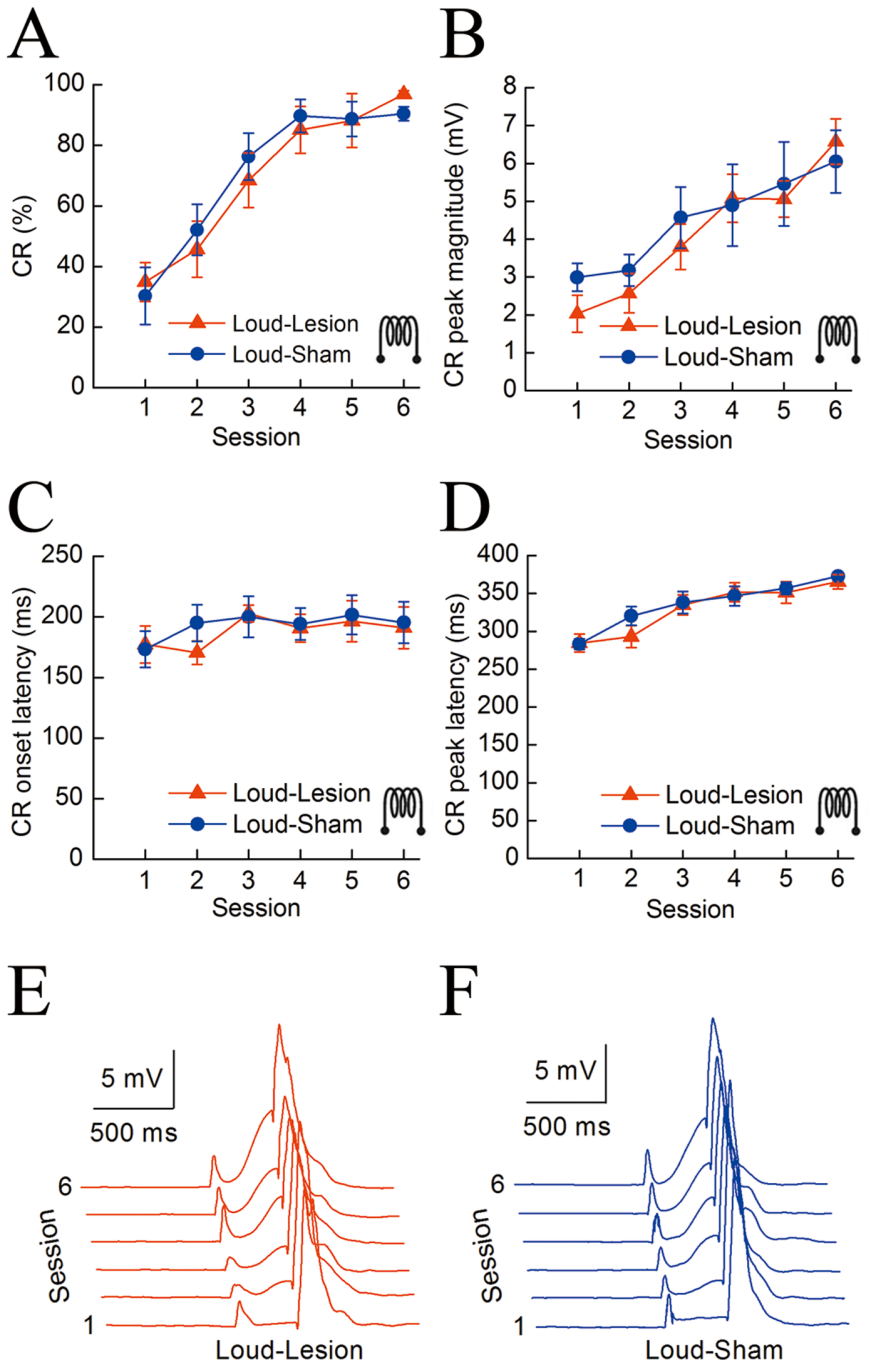
Effects of electrolytic lesions of the hippocampus before conditioning training on delay eyeblink conditioning with the loud tone CS. (A–D) The mean value ± standard error (SEM) for the percentage (A), peak magnitude (B), onset latency (C), and peak latency (D) of the CR with the loud tone CS in the Loud-Lesion (n = 7, red) and Loud-Sham (n = 7, blue) groups. (E, F) Averaged eyelid responses of all trials in the Loud-Lesion (E) and Loud-Sham (F) animals across six consecutive training sessions. Note that there were no significant lesion effects on the percentage (A), peak magnitude (B), onset latency (C), and peak latency (D) of the CR between the Loud-Lesion and Loud-Sham groups. The error bars represent the SEM.

It is worth asking whether the hippocampal lesions affect DEC with the loud tone CS. To this end, we investigated the effects of electrolytic lesions of the bilateral hippocampus before conditioning training on the DEC with the loud tone CS. Figure 5 shows the effects on the DEC with the loud tone CS of the electrolytic lesions and sham lesions of the bilateral hippocampus before the conditioning training in the Loud-Lesion (n = 7) and Loud-Sham (n = 7) groups. The data from one animal in the Loud-Lesion group and one animal in the Loud-Sham group were removed from the analysis because they died before the end of the experiment. As expected, a two-way repeated measures ANOVA on the CR% (Figure 5A) confirmed that there was no significant group by session interaction [*F*(5,60) = 0.365, *p* = 0.64] and no significant group effect [*F*(1,12) = 0.032, *p* = 0.860], but there was a significant session effect [*F*(5,60) = 45.285, *p*<0.001].

Similarly, to investigate the effects of the electrolytic lesions of the bilateral hippocampus on the pattern of the CR with the loud tone CS, we analyzed the CR peak magnitude, onset latency, and peak latency for these animals. There were no significant differences in CR peak magnitude, onset latency, or peak latency between the Loud-Lesion and Loud-Sham groups [a two-way repeated measures ANOVA; *F*(1,12) = 0.002, *p* = 0.966; *F*(1,12) = 0.105, *p* = 0.752; and *F*(1,21) = 0.230, *p* = 0.640; respectively], as depicted in Figure 5B–D.

### Effects of the Infusion of Muscimol into the Hippocampus on the Delay Eyeblink Conditioning with a Loud Tone CS

**Figure 6:**
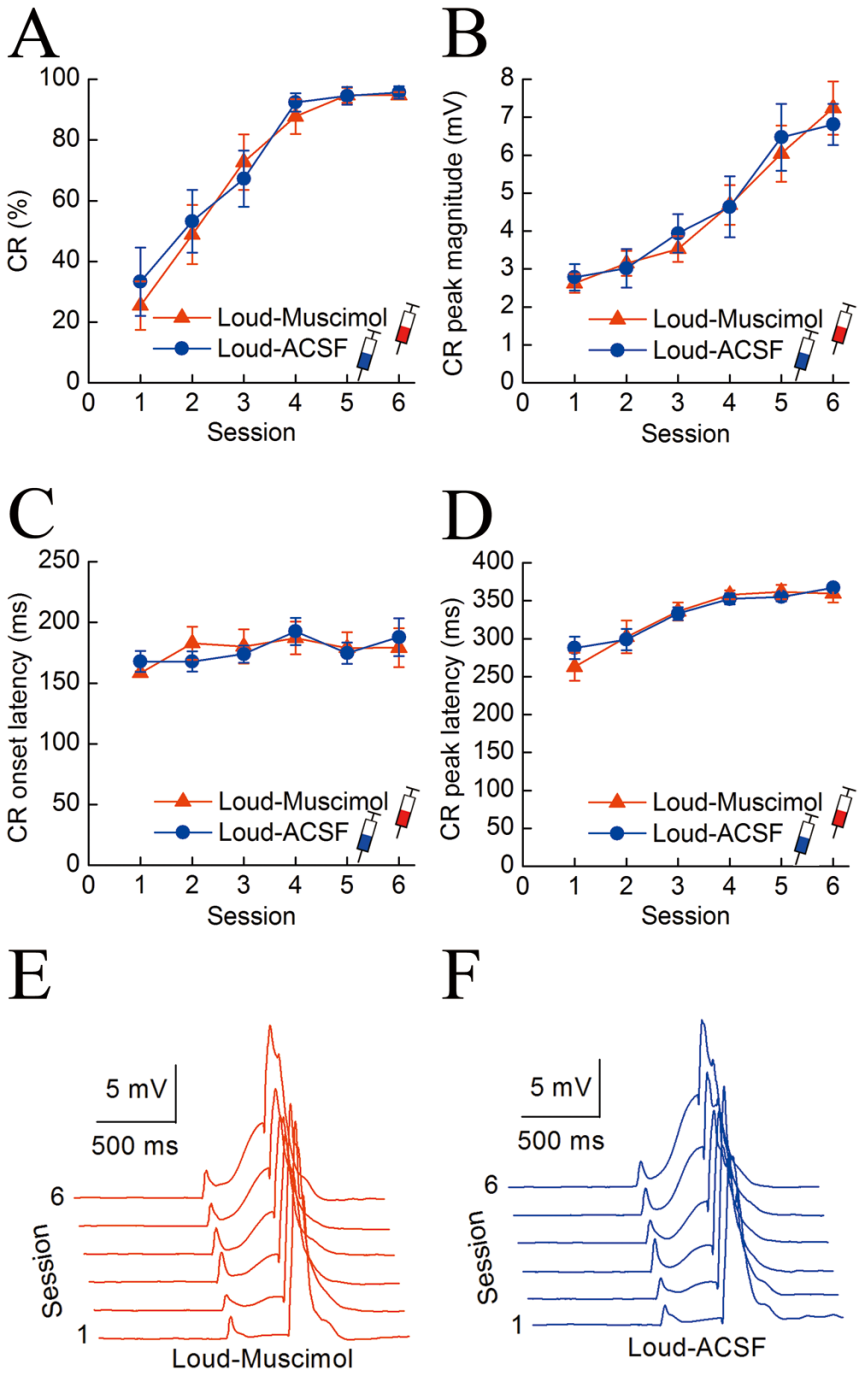
Effects of the infusion of muscimol into the hippocampus 30 min before daily conditioning training on delay eyeblink conditioning with the loud tone CS. (A–D) The mean value ± standard error (SEM) for the percentage (A), peak magnitude (B), onset latency (C), and peak latency (D) of the CR with the loud tone CS in the Soft-Muscimol (n = 8, red) and Soft-ACSF (n = 8, blue) groups. (E, F) Averaged eyelid responses of all trials in the Soft-Muscimol (E) and Soft-ACSF (F) animals across six consecutive training sessions. Note that there were no significant muscimol infusion effects on the percentage (A), peak magnitude (B), onset latency (C), and peak latency (D) of the CR between the Loud-Lesion and Loud-Sham groups. The error bars represent the SEM.

It is also worth asking whether the hippocampus is involved in other mnemonic processes (e.g., expression) of the delay CR with the loud tone CS. We investigated the effects of infusion of muscimol into the bilateral hippocampus 30 min before daily training during the inactivation phases (phases I and III) on the DEC with the loud tone CS. Figure 6 shows the effects on the DEC with the loud tone CS of the infusion of muscimol or ACSF into the bilateral hippocampus before daily conditioning training in the Loud-Muscimol (n = 8) and Loud-ACSF (n = 8) groups. As expected, a two-way repeated measures ANOVA on the CR% (Figure 6A) confirmed that there was no significant group by session interaction [*F*(5,70) = 0.454, *p* = 0.895], and no significant group effect [*F*(1,14) = 0.001, *p* = 0.983], but there was a significant session effect [*F*(5,70) = 41.036, *p*<0.001].

Similarly, to investigate the effects of the infusion of muscimol into the bilateral hippocampus on the pattern of the CR with the loud tone CS, we analyzed the CR peak magnitude, onset latency, and peak latency for these animals. There were no significant differences in CR peak magnitude, onset latency, or peak latency between the Loud-Muscimol and Loud-ACSF groups [a two-way repeated measures ANOVA; *F*(1,12) = 0.001, *p* = 0.995; *F*(1,12) = 0.002, *p* = 0.977; and *F*(1,21) = 0.35, *p* = 0.854; respectively], as shown in Figure 6B–D.

## Discussion

We set out to test our original hypothesis that the hippocampus is essential for the DEC with the soft tone CS but not for the DEC with the loud tone CS. The hypothesis appears to be supported by the present data because of the following findings: (a) the acquisition of the CR with the soft tone CS was significantly impaired by hippocampal lesions before training; (b) similarly, reversible inactivation of hippocampus caused significant deficits in both the acquisition and expression but not in consolidation of CR with the soft tone CS; and (c) both electrolytic lesions and reversible inactivation of hippocampus had no significant effects on the CR with the loud tone CS. It is important to note that the guinea pigs in Soft-Lesion and Soft-MAMA groups were not able to overcome the impairment with extensive training (2000 trials). These data, thus, strongly support our original hypothesis and suggest that the hippocampus plays an essential role specifically in the acquisition and expression of the delay CR with the soft tone CS. Together, these data argue against the prevailing idea that the hippocampus is not necessary for DEC. The present results on the DEC with the loud tone CS are in agreement with other studies [11]–[14].

Why did lesions of the hippocampus impair the DEC with the soft but not with the loud tone CS? Because the DEC with the soft CS is a more difficult task to acquire than DEC with the loud CS, and the hippocampus is involved in mediating the difficult task. Indeed, Beylin et al. [11] have reported that hippocampal lesions impaired acquisition of long DEC with 1400-ms ISI, but did not affect acquisition of DEC with 750-ms ISI, and suggested that the hippocampus is involved in the DEC when the task is sufficiently difficult to acquire. Moreover, the present data showed that the guinea pigs acquired the DEC with the soft tone CS at a much slower rate than they did DEC with the loud tone CS. Thus, the DEC with the soft CS is a more difficult task to acquire than DEC with the loud CS might explain why did lesions of the hippocampus impair the DEC with the soft but not with the loud tone CS. Alternatively, the soft tone CS would not activate mossy fibers sufficiently and DEC would only be possible by sustaining mossy fiber activity with hippocampus input. It has been proposed that the long-term depression (LTD) at the parallel fiber-Purkinje cell synapses in the cerebellar cortex and the long-term potentiation (LTP) at the mossy fiber synapses in the cerebellar deep nuclei are necessary for both the acquisition and expression of the CR [6], [42]–[46]. Moreover, cerebellar LTD and LTP at these synapses require an approximate time overlap of mossy fiber and climbing fiber activity [6], [45], [47]. However, only a small percentage of auditory-driven mossy fibers can show sustained responses that persist until the tone CS offset even when a loud tone CS is used [48]–[50], therefore, it can be speculated that the soft tone CS may drive much less mossy fibers activity, persisting until the tone offset. Thus, the cerebellar LTD and LTP at these conditions would be induced more difficultly by the soft tone CS relative to the loud tone CS. Although the sustained mossy fiber responses to the tone CS may also be potentially driven by input from sources such as the mPFC [37], inferior colliculus [51], or auditory thalamus [4], [5], [52]–[54], without hippocampus input, these activated mossy fibers alone may be insufficient to support the cerebellar LTD and LTP at these synapses during DEC with the soft tone CS. Thus, it is acceptable that lesions of the hippocampus impaired the DEC with the soft but not with the loud tone CS.

It is of special note that electrolytic lesions destroy not only the neurons within the radius of the lesion electrode but also the fibers of passage [16]. Thus, the deficits in DEC with the soft tone CS after hippocampal electrolytic lesions may result from the destruction of the output or input fibers that travel to or from other essential brain structures and may not be the result of a loss of neurons in the hippocampus. Thus, to specify the involvement of hippocampus in the memonic processes of the DEC with the soft tone CS, the present study also used muscimol infusion, which produces reversible inactivation only to the soma of hippocampus neurons but not to the fibers of passage. The reversible inactivation of the hippocampus caused significant deficits in both the acquisition and expression but not in consolidation of DEC with the soft tone CS. Furthermore, the data of phases I and II (Figure 4A) suggest that the acquisition of CR with the soft tone CS also occurred partially when muscimol was infused, because the CR% of Soft-MAMA group on the first session of phase I (session 1) was lower than that of Soft-MAMA group on the first session of phase II (session 21). The parsimonious interpretation of the present data is that the hippocampus may also play a critical role in modulating and/or sustaining the mossy fiber activity to overlap with the US during DEC with the soft tone CS. However, this hypothesis requires further testing.

It is apparent that the hippocampus is especially essential for DEC with the soft tone CS, because none of animals with the electrolytic lesions or muscimol inactivation of hippocampus was able to acquire the DEC with the soft tone CS even after 2000 trials training. However, the decerebrate guinea pigs even can successfully establish TEC with a trace interval of 500 ms after 2000 trials training [55]. It is unlikely that the soft tone CS is not a sufficient and effective CS for eyeblink conditioning in guinea pigs, because the animals of Soft-sham and Soft-AAAA groups have successfully acquired the DEC with the soft tone CS after 2000 trials training. It could be argued that the DEC with the soft tone CS used here is a more difficult task than TEC with a trace interval of 500 ms for guinea pigs. In addition, our findings extend those of Tam et al. [56], in which the effect of the ISI on delay appetitive conditioning in rats with hippocampal lesions was investigated. They reported that hippocampal lesions produced deficits in delay appetitive conditioning when the ISI was relatively long (40 s). The data combined with these findings suggest that brain areas responsible for one kind of classical Pavlovian conditioning paradigm may provide a similar function during other forms of classical Pavlovian conditioning.

In the TEC, it has been suggested that the cerebellum and hippocampus first form a short-term memory storage circuit, and as time goes on, long-term memory storage circuit increasingly depends on the cerebellum and mPFC [20], [57]. Simon et al. [15] proposed that permanent storage of trace CR is located in the deep nuclei of the cerebellum. Further, behavioral researches have demonstrated that increasing the difficulty of the DEC by extending the ISI can cause the result that hippocampal lesions impaired the long DEC [11]. Some recent studies also have shown that lesions of the mPFC affect TEC more when the US is a less salient airpuff than a more salient periorbital shock [17], [58], [59]. Our results combined with these findings suggest that the hippocampus and mPFC may play even greater roles in eyeblink conditioning when memory and attention demands are increased. The results of the present and previous [37] studies refine the understanding of the underlying role of the hippocampus in DEC. However, the underlying mechanisms of the hippocampus and mPFC involvement in the TEC versus DEC require further study.

In conclusion, the results from this study provide the first hand evidence that hippocampus is critical for DEC with a soft tone CS. Furthermore, we present data consistent with the view that hippocampus is not involved in DEC with a loud tone CS. We illustrate that the hippocampus is essential for the DEC when the cognitive task demands increase.
